# Evaluation of Risk Behavior in Gambling Addicted and Opioid Addicted Individuals

**DOI:** 10.3389/fnins.2020.597524

**Published:** 2021-01-07

**Authors:** Edward J. Gorzelańczyk, Piotr Walecki, Monika Błaszczyszyn, Ewa Laskowska, Aleksandra Kawala-Sterniuk

**Affiliations:** ^1^Department of Theoretical Basis of Bio-Medical Sciences and Medical Informatics, Nicolaus Copernicus University – Collegium Medicum, Bydgoszcz, Poland; ^2^Institute of Philosophy, Kazimierz Wielki University, Bydgoszcz, Poland; ^3^Babinski Specialist Psychiatric Healthcare Center, Outpatient Addiction Treatment, Lodz, Poland; ^4^The Society for the Substitution Treatment of Addiction “Medically Assisted Recovery”, Bydgoszcz, Poland; ^5^Department of Bioinformatics and Telemedicine, Jagiellonian University – Collegium Medicum, Krakow, Poland; ^6^Faculty of Physical Education and Physiotherapy, Opole University of Technology, Opole, Poland; ^7^Faculty of Medicine, Nicolaus Copernicus University – Collegium Medicum, Bydgoszcz, Poland; ^8^Faculty of Electrical Engineering, Automatic Control and Informatics, Opole University of Technology, Opole, Poland

**Keywords:** addiction, methadone therapy, gambling, opioids, Iowa gamble task, cortico-subcortical loops, philosophy of mind

## Abstract

Evidence suggests that both opioid addicted and gambling addicted individuals are characterized by higher levels of risky behavior in comparison to healthy people. It has been shown that the administration of substitution drugs can reduce cravings for opioids and the risky decisions made by individuals addicted to opioids. Although it is suggested that the neurobiological foundations of addiction are similar, it is possible that risk behaviors in opioid addicts may differ in detail from those addicted to gambling. The aim of this work was to compare the level of risk behavior in individuals addicted to opioid, with that of individuals addicted to gambling, using the Iowa Gambling Task (IGT). The score and response time during the task were measured. It was also observed, in the basis of the whole IGT test, that individuals addicted to gambling make riskier decisions in comparison to healthy individuals from the control group but less riskier decisions in comparison to individuals addicted to opioids, before administration of methadone and without any statistically significant difference after administration of methadone—as there has been growing evidence that methadone administration is strongly associated with a significant decrease in risky behavior.

## 1. Introduction

The evidence, collected by inter alia authors of this paper, suggests that both opioid addicted and gambling addicted individuals can be characterized with higher levels of risk behavior in comparison to healthy people (Brevers et al., 2013; Gorzelańczyk et al., 2014). It has been shown that the administration of substitution drugs can reduce cravings for opioids and also decrease risky decision making among individuals addicted to opioids (Gorzelańczyk et al., 2014; Hu et al., 2019; Kriegler et al., 2019; Kelty et al., 2020). Although it is suggested that the neurobiological basis of addiction is similar, it is possible to assume that the risk behavior in individuals addicted to opioids can differ from gambling addicted individuals (Zeng et al., 2016; Chen et al., 2017; Coppola et al., 2017; Majuri et al., 2017; Schwaninger et al., 2017; Victorri-Vigneau et al., 2018; Kim et al., 2019).

Therefore, the main aim of this study was to compare the level and dynamics of risk behavior in opioid addicts with those addicted to gambling while performing the Iowa Gambling Task (IGT). The score and response time were measured during the IGT performance. The authors introduced for the first time the response time measurement in the IGT test as a new parameter. Response time is assessed for correct and incorrect choices and can be useful in the differential diagnosis of addicts Gorzelańczyk et al. (2014).

A large number of similarities between drug addiction and gambling addiction were found recently. It was noticed that those addictions share some common mechanism (American Psychiatric Association, 2013; Brevers et al., 2013). Addicted individuals are more prone to show risky behavior in comparison to healthy people (Leeman and Potenza, 2012; Ahmadi et al., 2017; Chamberlain and Grant, 2019).

It was also observed that impairments in decision-making reflect drifts into risky behaviors and may be at first manifested with some psychiatric symptoms or cognitive dysfunctions (Chamberlain and Grant, 2019; Vegni et al., 2019). Misuse and addiction to opioids have become a major civil challenge in the world (Volkow et al., 2018).

Gambling is an activity, where something valuable is risked on behalf of a chance for winning something even more valuable (Yau and Potenza, 2015). The chances are however less than certain (Nautiyal et al., 2017). At first it may seem like a recreational activity, as between 50 and 80% of the general population gamble at least once a year. Individuals who are addicted to gambling tend to increase their risky behavior, which can result in serious financial problems (Leeman and Potenza, 2012; Brevers et al., 2013).

From the neuropsychiatric and neurobiological perspectives, risky behavior is connected to malfunctioning of mesolimbic and executive control circuits (American Psychiatric Association, 2013; Engel and Caceda, 2015). It is known that the use of psychoactive substances can change structures and functions of circuits involved in risk decision making (Gilman et al., 2015). The structural and functional changes of the elements of the cortico-subcortical loops have been observed among addicted people (Gorzelańczyk et al., 2014, 2016; Tarnowska et al., 2018).

It has also been observed that poker gamblers exhibited higher ventral-striatal but lower dorsolateral prefrontal and orbitofrontal activation during Iowa Gambling Task performance as well as higher ventral-striatal connectivity and connectivity in posterior cingulate cortex, occipital gyrus, and temporal gyrus (Leeman and Potenza, 2012). In addition, the severity of gambling is associated with the activation of ventral striatum, occipital fusiform gyrus and middle temporal gyrus (Brevers et al., 2016). The data from experiments on pathological gamblers show increased activation in response to visual gambling cues in such brain structures as the right dorsolateral prefrontal cortex, the right parahippocampal gyrus, and the left occipital cortex (Leeman and Potenza, 2012; Epstein and Silbersweig, 2016; Chamberlain and Grant, 2019).

In this paper the authors compared various IGT parameters of risky behaviors between addicted (to gambling or opioids) and healthy individuals using the most popular Iowa Gambling Task, which is still regarded as the classical measurement tool for decision making in this clinical population (Brevers et al., 2013). The IGT test is used to assess risky behavior also in addicted individuals (Ahn et al., 2016b; Mallorquí-Bagué et al., 2016; Kovács et al., 2017; Brière et al., 2019; Khoury et al., 2019; Lin et al., 2019; Trotzke et al., 2019).

### 1.1. IOWA Gambling Task

The Iowa Gambling Task (IGT) is a psychological test with a continuous task performed on a computer, which simulates various situations for decisions making (Bechara et al., 1994).

Disease categories associated with risky behaviors include inter alia: compulsive stealing (kleptomania), compulsive shopping, and compulsive sexual behavior as well as addictions to opioids and other chemical substances (Chamberlain and Grant, 2019).

The Iowa Gambling Task (IGT) is the most popular test for assessment of an appropriate decision-making process (Bechara et al., 1994; Tanabe et al., 2007; Brevers et al., 2013). It deals with uncertainty in a context of penalty and reward, with some choices being advantageous in the short-term (high reward), but disadvantageous in the long run (higher penalty), there are also other choices, which are less attractive in the short-term (low reward), but advantageous in the long run (lower penalty) (Brevers et al., 2013; Vasconcelos et al., 2015; Smith et al., 2016). It shows preferences of the tested participants for choosing short-term gains at the risk of larger loses (Tanabe et al., 2007). The choice between long- and short-term benefits enables distinction between gambling and opioid addicts and description of particular decision-making mechanisms. Also, IGT can be a very good measure of impaired decision-making in people suffering various psychiatric or neurological conditions (Bechara et al., 1994; Upton et al., 2012; Brevers et al., 2013). The IGT is the most popular decision-making task applied in numerous clinical studies (Upton et al., 2012; Ahn et al., 2016a).

### 1.2. Background to the Study

Studies on decision making in addicted participants have a very long history and have resulted in the common knowledge that substance addicted individuals (SDI) usually prefer choices bringing immediate benefits, even if there are negative consequences, such as inter alia loss of job, home or family. For such study purposes the IGT simulating real life decision making is frequently carried out. It is also the most popular decision-making task that has been applied in numerous clinical studies (as it was mentioned above) (Upton et al., 2012).

Based on a thorough literature background—both drug addicts and healthy study participants tend to choose decks with net losses at the beginning of the test, but only the healthy individuals are able to shift their choices to the decks with net gains, learning from their experience, while the addicted individual fail to do so (Bechara and Damasio, 2002; Bechara et al., 2002; Upton et al., 2012).

## 2. Methods

**Figure 1:**
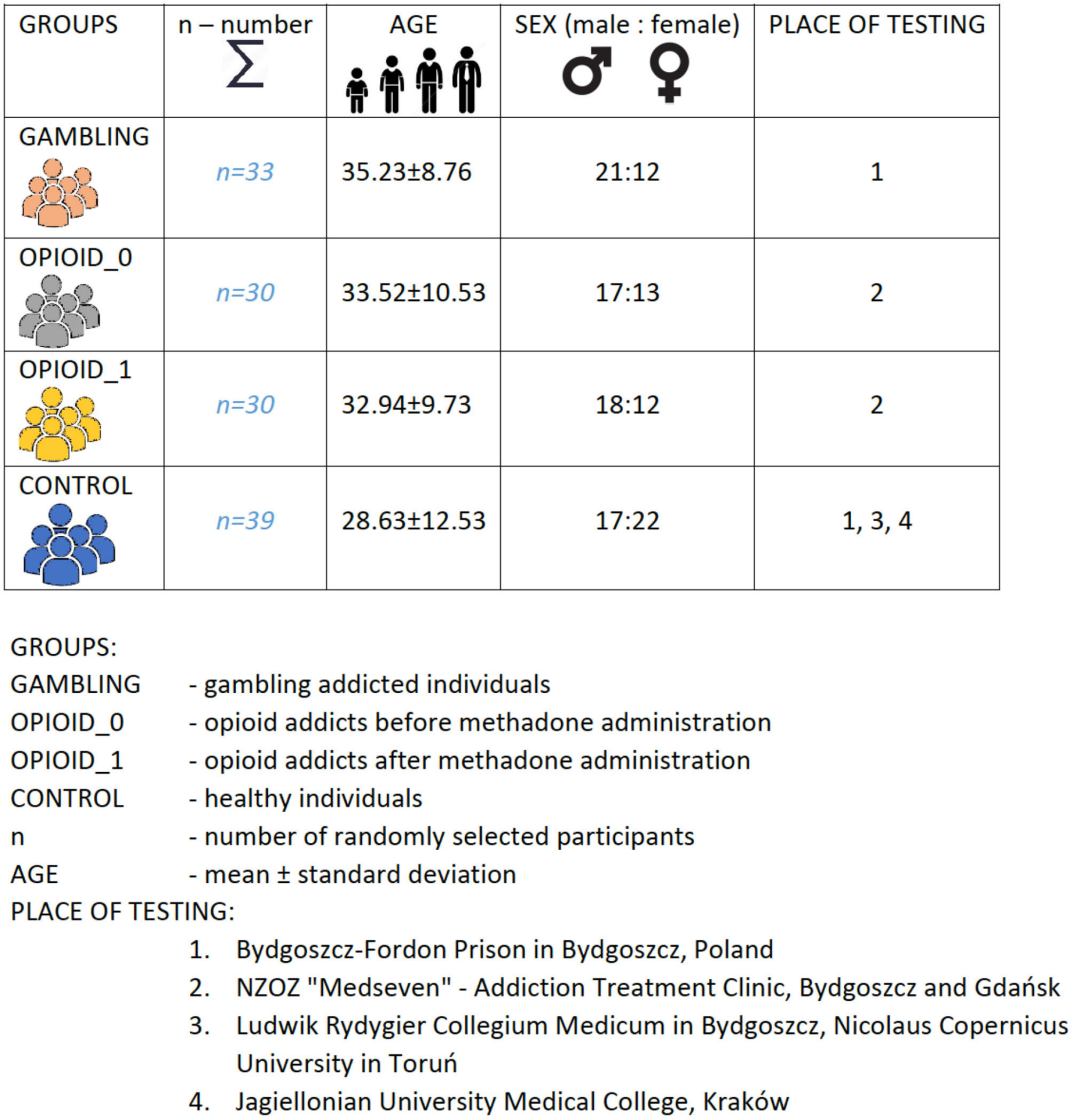
Study participants—summary.

For the study purposes 132 subjects (***n*** **=** **132**) were recruited for this study from opioid substitution clinics in various towns in Poland (Bydgoszcz, Gdańsk, Kraków). Summary of study-participants was illustrated with Figure 1.

The participants were recruited among clinics' patients, prisoners, and healthy people. The participants were diagnosed in accordance with the *ICD*−10 criteria.

The participants' selection criteria included:

- Meeting the diagnostic and statistical manual criteria of opioid dependence;
- Age range 21–60 years old;
- Absence of illicit drugs or alcohol withdrawal or intoxication at time of the study visits;
- Absence of history of psychotic mental illnesses or history of traumatic brain injuries;
- Absence of history of cognitive or memory problems;
- Subjects are stable on methadone maintenance for at least 2 weeks.

The recruitment of gambling addicts was carried out mainly in prisons. The male/female ratio among gambling addicts is higher among males, as female prisoners constitute only 4.4% of all prisoners in Poland (2020). Gender gambling addiction research requires additional logistical efforts due to the significant disproportion in the frequency of this disorder between males and females. One-sex comparative studies are advisable.

The Mini-Mental State Examination (MMSE) was used as a screening device for cognitive impairment. The individuals with a minimum of 27 points in MMSE were included in the study. The educational structure of the particular group is presented in Table 1.

**Table 1:** Structure of education among investigated individuals.

	**Gambling**	**Opioid**_**0**	**Opioid**_**1**	**Control**
Level of education	*n* = 33	*n* = 30	*n* = 30	*n* = 39
Primary	18	20	19	17
Secondary	10	8	8	15
Tertiary	5	2	3	7

The gambling addicts were included in the study after a psychiatric interview examination. Nicotine addiction did not exclude individuals from the study. Individuals with comorbid psychiatric disorders other than gambling were however excluded.

The dose of this substitution was selected individually in order to prevent the occurrence of withdrawal symptoms. The average dose of methadone in the study group was 70 mg per day, administered orally in a single dose.

The limitation concerning the level of education of the study participants results from sources of acquired material. However, the behavioral strategy in performing the IGT test is quite specific for gambling addicts. Further research is necessary in terms of gender and education.

All participants (opioids addict, gambling addicts, healthy individuals) had to provide written informed consent in order to take part in this study. To conduct the study, the consent of the Bioethics Commission at Medical College in Bydgoszcz, Nicolaus Copernicus University in Torun, Poland was obtained (Consent no. *KB*/416/2008).

After being appropriately classified for the project-participation—each subject was scheduled for the IGT session testing. The opioid addicted individuals were divided into two groups. Individuals from the first group were tested before the methadone administration and individuals from the second group performed the IGT test about one and a half hours after the methadone administration.

After consenting to participate in the study, each subject was scheduled for the sessions of IGT testing. The IGT is a psychological task thought to simulate real-life decision making (Bechara et al., 1994). The authors of this work used the IGT, which is a part of the PEBL Test Battery (Mueller and Piper, 2014).

The construction of the IGT test consists of simulation games and gambling. In the task there are four decks of cards which contain the winner's and loser's cards. The winner's and loser's cards contain different monetary values. It means that for each deck of cards a certain amount of reward and penalty is attributed. An individual has to choose a deck containing the cards of the highest profit (Bechara et al., 1994; Fineberg et al., 2010). There are the four decks of cards marked as *A*, *B*, *C*, and *D*. The first two decks (*A* and *B*) are disadvantageous since, although immediate gains are large, the gain is followed by large losses at unpredictable intervals. In contrast, the other two decks (*C* and *D*) are advantageous.

In this case the immediate gains are smaller but there are also unpredictable losses, which are also small so that in the longer term the player gains more.

During the IGT test performance, the study subjects sit in front of a computer screen. The study is carried out in a way that, while using the computer's mouse, the study participant clicks on a card from any of the four decks. A standard administration of 100 trials (i.e., selection of 100 cards) was done once in individuals from all groups (opioid addicted, behaviorally addicted and healthy individuals). In this study the response time was given in milliseconds (ms) and for every subject was recorded in every trial. The response time is defined as time from the appearance of the cards on the screen till the time of card selection (by clicking a computer mouse button). The main dependent measure used for the calculation of the IGT performance was the net score. It was calculated by subtracting the number of cards selected from the disadvantageous decks from the numbers of cards selected from the advantageous decks (*C*+*D*)−(*A*+*B*). Lower scores reflected a more disadvantageous than advantageous decision-making performance.

**Figure 2:**
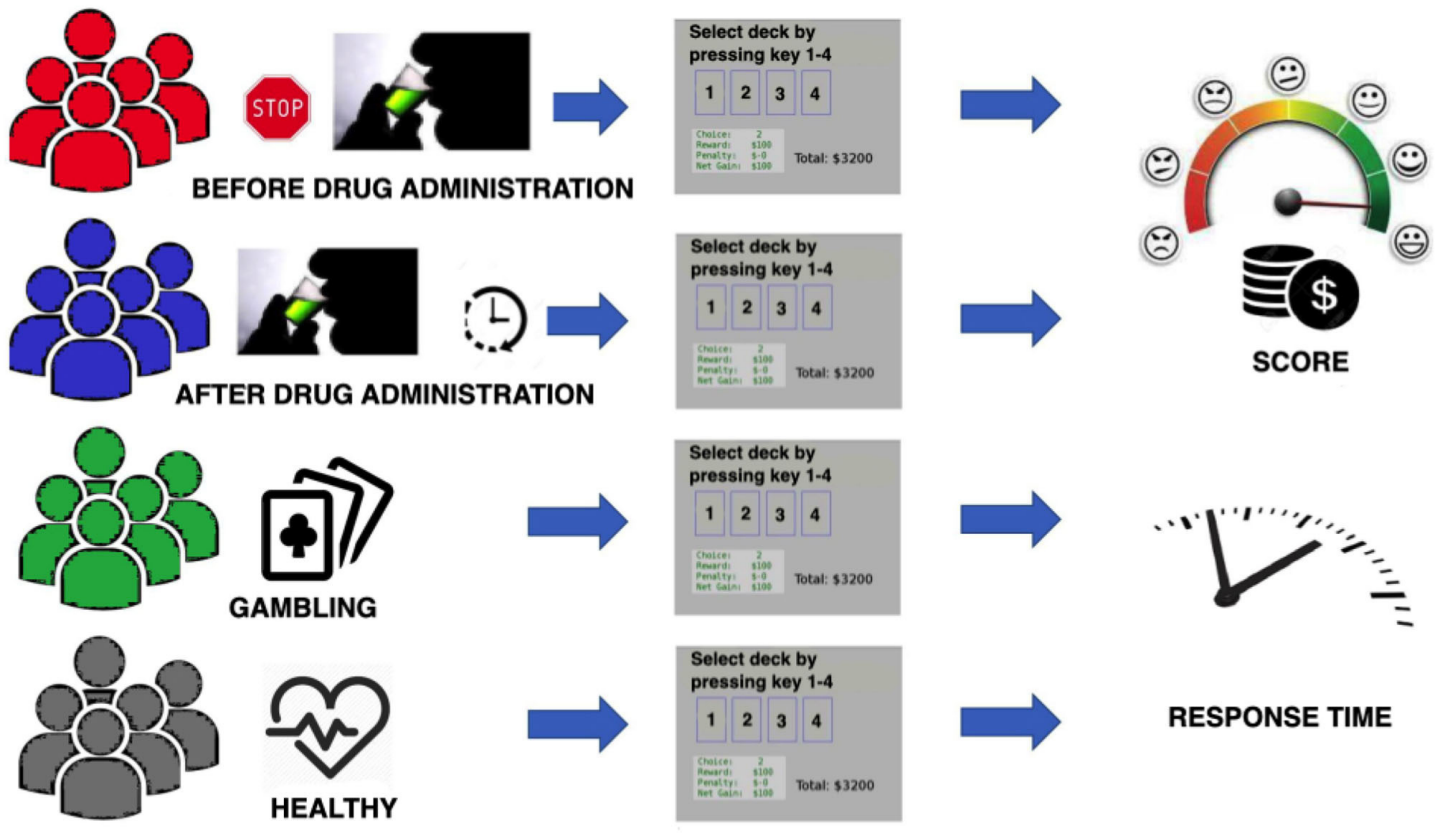
General scheme of the conducted experiment.

In Figure 2, a general scheme of the conducted experiment is presented. Individuals from each group were tested once. This procedure excludes the learning effect Pasion et al. (2017); Almy et al. (2018).

## 3. Results

In this work the number of risky decisions (decks A + B) and safe decisions (decks C + D) was compared in opioid addicts (before and after methadone administration) with those addicted to gambling and with the healthy individuals.

It was found (One-way ANOVA. *F* = 4.529, *p* = 0.00472) that the participants from the control group 34.85 ± 11.52 take less risky decisions compared to the gambling addicts 41.85 ± 11.05 (mean and standard deviation) and to the opioid addicted individuals (before the administration of methadone) 46.07 ± 15.09 and to the opioid addicted individuals after the methadone administration 42 ± 14.46.

It was also found (One-way ANOVA *F* = 6.666, *p* = 0.00032) that the gambling addicted individuals are less likely to make safe decisions (58.152 ± 11.0542) compared to the subjects from the control group (65.359 ± 11.5656), but take less safe decisions compared to the opioid addicted individuals prior the methadone administration (51.767 ± 14.0263) and that there is no significant statistical difference compared to the opioid addicted individuals after the methadone administration (57.6 ± 14.2577). In Table 2 (for Risky Decisions) and Table 3 (for Safe Decisions) the obtained results using Turkey HSD test are presented.

**Table 2:** Values for probabilities after *post-hoc* tests—risky decisions.

	**Turkey HSD test; variable risky decisions**.				
	**Approximate probabilities for** ***post-hoc*** **tests;**				
	**Error: between MS** **=** **168.84, ***df*** = 128.00**				
		**(1)**	**(2)**	**(3)**	**(4)**
**Cell no**.	**Groups**	**41.848**	**34.846**	**46.067**	**42.000**
1	Gambling		**0.046465**	0.457858	0.999948
2	Control	**0.046465**		**0.000300**	**0.048094**
3	Opioid_0	0.457858	**0.000300**		0.511049
4	Opioid_1	0.999948	**0.048094**	0.511049	

*Bold values are significantly important statistical values (p < 0.05)*.

**Table 3:** Values for probabilities after *post-hoc* tests—safe decisions.

	**Tukey HSD test; Variable Safe Decisions**.				
	**Approximate Probabilities for** ***post-hoc*** **Tests;**				
	**Error: Between MS** **=** **160.89, ***df*** = 128.00**				
		**(1)**	**(2)**	**(3)**	**(4)**
**Cell no**.	**Groups**	**58.152**	**65.154**	**51.767**	**57.600**
1	Gambling		**0.049168**	0.140795	0.997764
2	Control	**0.049168**		**0.000020**	**0.034958**
3	Opioid_0	0.140795	**0.000020**		0.223987
4	Opioid_1	0.997764	**0.034958**	0.223987	

*Bold values are significantly important statistical values (p < 0.05)*.

It was found (ANOVA *F* = 14.164, *p* = 0.00000) that the average response time (milliseconds) of gambling addicts (1516.748 ± 930.15) was statistically significantly longer than the mean response time of the individuals from the control group (646.6121 ± 284.23) and had a similar mean response time without a significant statistical difference when compared to the opioid addicted individuals prior to methadone administration (1418.943 ± 823.46) and the opioid addicts after the administration of methadone (1654.676 ± 757.99).

It was also found that the mean response time of the individuals from the control group after reward 636.641 ± 273.872 and after penalty 682.8462 ± 351.07 (Wilks lambda *F* = 8.856, *p* = 0.000) is statistically significantly shorter than the mean response time of gambling addicts after reward (1580.636 ± 965.5572) and penalty (1296.030 ± 910.7465), opioid addicts before the methadone administration (reward 1680.567 ± 840.7316; penalty 1530.933 ± 845.7030) and opioid addicts after taking methadone (reward 1432.9 ± 760.0489; penalty 1357.167 ± 794.7040).

In Table 4, the response time (expressed in [ms]) after reward, as a result of *post-hoc* tests is presented, while Table 5 presents the response time after penalty.

**Table 4:** Values for the response time (in [ms]) after *post-hoc* tests—after reward.

	**Tukey HSD test; Variable response time [ms] after reward**.				
	**Approximate probabilities for** ***post-hoc*** **tests;**				
	**Error: between MS** **=** **5464***E***2, ***df*** = 128.00**				
		**(1)**	**(2)**	**(3)**	**(4)**
**Cell no**.	**Groups**	**1580.6**	**636.64**	**1680.6**	**1432.9**
1	Gambling		**0.000008**	0.950305	0.857985
2	Control	**0.000008**		**0.000008**	**0.000060**
3	Opioid_0	0.950305	**0.000008**		0.564378
4	Opioid_1	0.857985	**0.000060**	0.564378	

*Bold values are significantly important statistical values (p < 0.05)*.

**Table 5:** Values for the response time (in [ms]) after *post-hoc* tests—after penalty.

	**Tukey HSD test; variable response time [ms] after penalty**.				
	**Approximate probabilities for** ***post-hoc*** **tests;**				
	**Error: between MS** **=** **5491***E***2, ***df*** = 128.00**				
		**(1)**	**(2)**	**(3)**	**(4)**
**Cell no**.	**Groups**	**1296.0**	**682.85**	**1530.9**	**1357.2**
1	Gambling		**0.002647**	0.590658	0.987930
2	Control	**0.002647**		**0.000021**	**0.001044**
3	Opioid_0	0.590658	**0.000021**		0.800470
4	Opioid_1	0.987930	**0.001044**	0.800470	

*Bold values are significantly important statistical values (p < 0.05)*.

**Figure 3:**
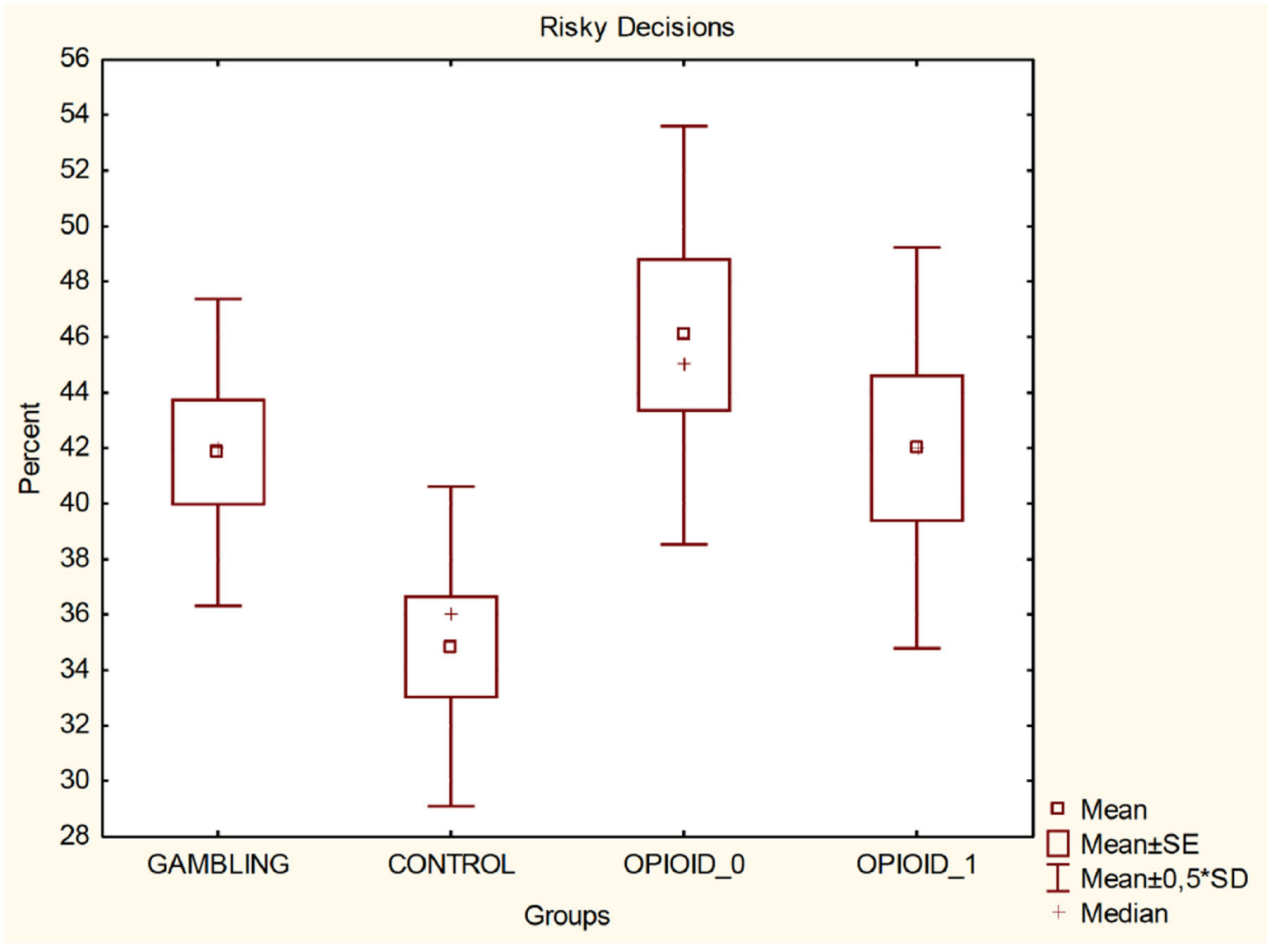
Risky decisions—percentage.

**Figure 4:**
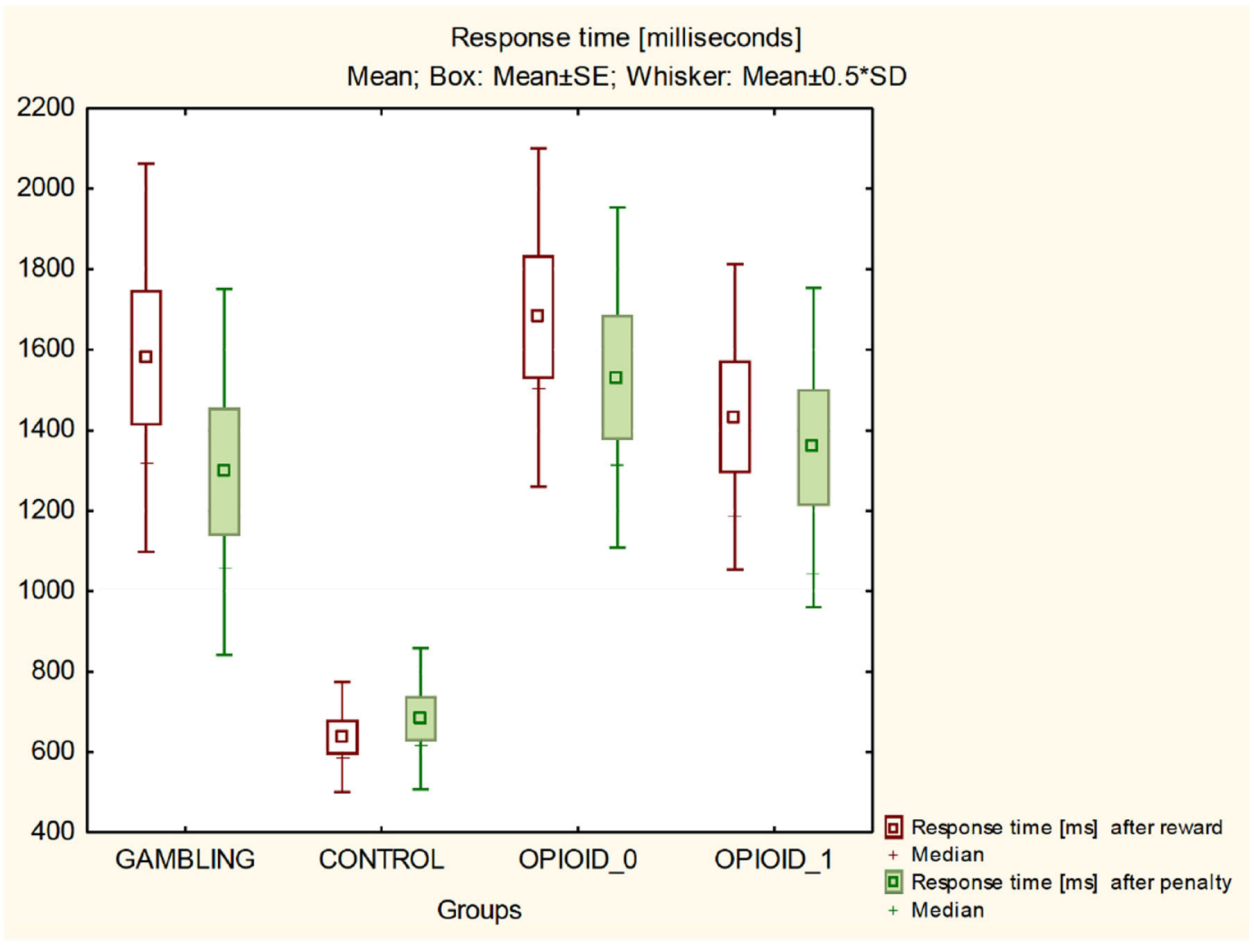
Response time after reward and penalty—comparison of all groups.

Figure 3 illustrates the percentage of made risky decisions. Figure 4 shows comparison of all groups in regards of time after reward and after penalty.

Table 6 presents values for the response time—mean of the entire test.

**Table 6:** Values for the response time [ms] mean of the entire test.

	**Tukey HSD test; variable response time [ms] after penalty**.				
	**Approximate probabilities for** ***post-hoc*** **tests;**				
	**Error: between MS** **=** **5241***E***2, ***df*** = 128.00**				
		**(1)**	**(2)**	**(3)**	**(4)**
**Cell no**.	**Groups**	**1516.7**	**646.61**	**1654.7**	**1418.9**
1	Gambling		**0.000010**	0.874445	0.950398
2	Control	**0.000010**		**0.000008**	**0.000072**
3	Opioid_0	0.874445	**0.000008**		0.587783
4	Opioid_1	0.950398	**0.000072**	0.587783	

*Bold values are significantly important statistical values (p < 0.05)*.

## 4. Discussion

Various cognitive models have been applied and developed for the past 20 years in order to understand decision making deficits in drug-addicted, brain-damaged individuals (Ahn et al., 2016a).

Decision making triggers simultaneous motor, emotional, and cognitive functions (Dixon et al., 2017; Weinstein et al., 2017; Hilber et al., 2019). Therefore, the authors of this work are looking for answers on to what extent behavioral and chemical addictions have common features and to what extent they differ from each other. The results of many studies indicate that alcohol, cocaine, heroin, cannabinoids, nicotine, and glucose as well as gambling increase risky behavior and cause activation and neuronal release of the brain dopaminergic system, which could heal the abnormal cravings (Blum et al., 2000; Anselme and Robinson, 2013).

These results indicate the importance of cortico-subcortical loops in decision making when performing an IGT test. Mesolimbic dopamine is the main transmitter in striatum that is released to a larger extent in pathological gamblers than in healthy individuals (Linnet et al., 2011, 2012; Potenza, 2018). The impulsive, addictive, and compulsive behaviors have common characteristics (Brevers et al., 2014; Lorains et al., 2014).

Individuals with Reward Deficiency Syndrome (RDS) differ with their reactions in comparison with healthy people. It has been assumed that there is a relationship between psycho-motor ability and decision making. The working hypothesis is that people who suffer from RDS process decisions differently than healthy people. The postulated reason for this difference is based on the observation that the activity of the limbic loop (which is responsible for the processing of emotions) in individuals with the impaired RDS (Fotros et al., 2013).

In contrast, it is interesting that people addicted to gambling make less risky decisions compared to opioid-dependent individuals before the administration of methadone.

Four independent groups were compared in these studies:

- Gambling addicts,
- Opioid addicts treated with methadone substitution before administration of this drug,
- Opioid addict treated with substitution after administration of methadone,
- Healthy individuals.

The Iowa Gambling Test was performed only once. This procedure excludes the learning effect (Pasion et al., 2017; Almy et al., 2018). Previous results of our own research indicate that administration of a substitution drug in opioid dependent individuals improves decision making.

It has been observed that the substitution drug (methadone) reduces the level of risky behavior in opioid addicts (Gorzelańczyk et al., 2014). It is interesting to see whether the decision-making strategy before administration of the substitution drug is similar in opioid addicts to the strategy in those who are addicted to gambling and whether the decision-making strategy after administration of the drug is similar to healthy individuals, which has been stated in inter alia: (King et al., 2015; Gallimberti et al., 2016).

To summarize the overall discussion, it is important to mention that a common neurobiological mechanism for chemical and behavioral addictions is still postulated, however, clinical observations and research results show some differences between different types of addiction (Jiang et al., 2020).

The IGT performance strategy is for gamblers and opioid dependent individuals without a similar substitution drug administration. But it is important to mention that gamblers' strategy leads to an endpoint similar to that of opioid addicts without administration of the drug (opioid).

The gamblers have the potential to learn from mistakes, but for some reason, during the IGT processing time, they stop learning. The explanation may be the strong activation of the striatum in gamblers at the beginning of the test, which results in the control of subcortical structures and the lack of effective inhibition of the striatum by the cerebral cortex.

Perhaps playing is much more important than winning for gamblers, as for them the game is a trigger, and it is difficult to stop the process. This is perhaps a similar mechanism to the one in alcoholics, thus gaming for gamblers is the same as an alcoholic going on a drinking binge (Cavicchioli et al., 2018).

The above mentioned mechanisms observed in gamblers are similar to the pattern followed by alcohol or drug addicted individuals.

Presumably, just joining the game temporarily increases the cognitive performance of gamblers. The IGT cues are consistent with gambling addiction and they easily fall into a binge. This is why gamblers quickly deplete cognitive resources due to the type of stimuli—although their absolute resources are greater compared to those addicted to opioids.

In opioid addicts, decision making also depends on the type of substitution drug used for substitution treatment (Pirastu et al., 2006). It was found that methadone administration is associated with impairment in the decision making ability but during dosage increase the decision making appears to improve (Barahmand et al., 2016).

These results indicate that stimulation of the reward system in both gambling addicts and opioid addicts is similarly difficult, and administration of substitution medication does not significantly reduce the response time of the opioid addicts.

It is possible to observe a significant decrease of scores and response times after penalty during the fourth deck in gambling individuals during the IGT task performance. This is assumed to be the consequence of control taking by subcortical structures and clear evidence of a reward system deficit, which results in particular decisions being taken by the addicted participants of this study and is impossible to observe in healthy, non-addicted individuals.

According to the authors' knowledge, no data are available in the literature characteristics for addicted individuals to drop at the fourth trial of the IGT. This result requires confirmation and further research.

Original results can also indicate that stimulation of the reward system in both gambling addicts and opioid addicts is similarly difficult, and administration of substitution medication does not significantly reduce the response time of opioid addicts. It is possible that the reward system is more difficult to stimulate in addicts in comparison to healthy people.

There are some limitations regarding gender ratio among the participants between the groups tested. In our original research it was found that gambling addiction is much more common among male compared to female among prisoners. However, the behavioral strategy in performing the IGT test is quite specific for gambling addicts. Further research is necessary in terms of gender and education. Also, the female prisoner ratio is significantly lower than male poland (2020).

Interestingly, the administration of opioid substitution drugs—methadone improves the performance of the IGT test (reduces the level of risky behavior) in opioid addicted individuals. Even though an improvement is observed during the IGT test performance in opioid addicted individuals after methadone administration, compared to the level of performance reached by gamblers, this improvement did not reach the level of performance observed in healthy, non-addicted individuals.

It is interesting that the average response time from noticing the reward to pressing the button is greater in gamblers in comparison to the response time of healthy individuals. This result may indicate that the activation of the reward system in gamblers is more difficult in comparison with the participants from the control group.

The results of the presented studies show that, in opioid addicts treated with methadone in the substitution treatment program, a single dose of methadone affects the number of risk behaviors measured by the IGT test.

The results of the presented studies indicate that, in opioid addict individuals treated with methadone in the substitution treatment program, a single dose of methadone affects the level of risk behaviors measured by the IGT test. It is possible that the risk behavior in individuals addicted to opioids can differ from gambling addicted individuals, despite some assumptions regarding similarity of both. The level of risky behavior in both addictions was compared during this study, including time-response during tasks. It was observed on the basis of the whole IGT test that gambling addicted individuals take more risky decisions in comparison to healthy individuals from the control group but less risky decisions in comparison to opioid addicted individuals before administration of methadone and without any statistically significant difference after administration of methadone. Various research and clinical observations postulate that there are both some similarities and some differences between drug addiction and behavioral addiction symptoms (Kluwe-Schiavon et al., 2019). Finding objective differences in behavior strategies can help to distinguish between substance—and gambling-addicts (Kriegler et al., 2019).

Addicted people more frequently display risky behavior. The analysis of respondents after the administration of methadone showed a statistically significantly decrease of the tendency to display risky behaviors. Results of studies (based on authors' experience and literature study) show that addicted people tend to display risky behaviors in subsequent attempts of the IGT test.

It is also important to consider individual differences in risk-tolerance, as these are crucial factors in taking risky decisions. Some dependency between obesity and various addictions (Yi et al., 2007; Rasmussen et al., 2010; Petry, 2012; Castren et al., 2015) was also found. Another meaningful factor is gender-based, as some studies show that men are more prone to addiction than women. Further studies in this area would be an advantage (Carneiro et al., 2019). Appropriate stimuli can affect decisions made for choosing the correct reward (Blanchard et al., 2015; Smith et al., 2016, 2017; Zentall, 2016).

The authors of this paper would like in the near future to investigate other pharmacological agents, such as inter alia: including serotonin reuptake inhibitors (SRIs), opioid antagonists, glutamatergic agents, and the anti-dopaminergic medication olanzapine, which gave (based on a literature study) promising results, as described in Chamberlain and Grant (2019).

It is also planned to differentiate the obtained results based on the gender of the tested individuals (Carneiro et al., 2019). There is a limitation regarding the level of education, resulting from the source of the acquired material. However, the behavioral strategy in performing the IGT test is quite specific for gambling addicts. Further research is necessary in terms of gender and education and should be expanded in further research plans.

## Data Availability Statement

The datasets used and analyzed during the current study will be made available by the corresponding author upon reasonable request.

## Ethics Statement

The studies involving human participants were reviewed and approved by Bioethics Commission at Medical College in Bydgoszcz, Nicolaus Copernicus University in Torun, Poland was obtained (Consent no. KB/416/2008). The patients/participants provided their written informed consent to participate in this study.

## Author Contributions

EG: designed the study protocol, recruited participants, carried out research, and prepared the draft version of the paper. PW: carried out research and analyzed the results. MB: performed deep literature study, partially wrote the Future Works section and the Discussion. AK-S: prepared the final version of the manuscript, expanded the draft version, performed a very thorough literature study, proof-read the article, prepared the template, and partially wrote the Discussion and Future Works sections. EL: helped with the patients recruitment and carried out the study. All authors contributed to the article and approved the submitted version.

## Conflict of Interest

The authors declare that the research was conducted in the absence of any commercial or financial relationships that could be construed as a potential conflict of interest.
